# Epidemiology of *Cryptosporidium* infection in cattle in China: a review

**DOI:** 10.1051/parasite/2017001

**Published:** 2017-01-18

**Authors:** Chao Gong, Xue-Feng Cao, Lei Deng, Wei Li, Xiang-Ming Huang, Jing-Chao Lan, Qi-Cheng Xiao, Zhi-Jun Zhong, Fan Feng, Yue Zhang, Wen-Bo Wang, Ping Guo, Kong-Ju Wu, Guang-Neng Peng

**Affiliations:** 1 The Key Laboratory of Animal Disease and Human Health of Sichuan Province, College of Veterinary Medicine, Sichuan Agricultural University Chengdu 611130 Sichuan PR China; 2 Chengdu Research Base of Giant Panda Chengdu 611130 Sichuan PR China; 3 Center for Disease Control and Prevention of Chengdu Military Region Kunming 650118 Yunnan PR China

**Keywords:** *Cryptosporidium* subtypes, Geographical distribution, Cattle, China, Zoonosis

## Abstract

The present review discusses the findings of cryptosporidiosis research conducted in cattle in China and highlights the currently available information on *Cryptosporidium* epidemiology, genetic diversity, and distribution in China, which is critical to understanding the economic and public health importance of cryptosporidiosis transmission in cattle. To date, 10 *Cryptosporidium* species have been detected in cattle in China, with an overall infection rate of 11.9%. The highest rate of infection (19.5%) was observed in preweaned calves, followed by that in juveniles (10.69%), postweaned juveniles (9.0%), and adult cattle (4.94%). The dominant species were *C. parvum* in preweaned calves and *C. andersoni* in postweaned, juvenile, and adult cattle. Zoonotic *Cryptosporidium* species (*C. parvum* and *C. hominis*) were found in cattle, indicating the possibility of transmission between humans and cattle. Different cattle breeds had significant differences in the prevalence rate and species of *Cryptosporidium*. This review demonstrates an age-associated, breed-associated, and geographic-related occurrence of *Cryptosporidium* and provides references for further understanding of the epidemiological characteristics, and for preventing and controlling the disease.

## Abstract (fr)

La présente étude discute les résultats de la recherche sur la cryptosporidiose menée chez les bovins en Chine et met en évidence les informations actuellement disponibles sur l’épidémiologie, la diversité génétique et la distribution en Chine de *Cryptosporidium*, essentielles à la compréhension de l’importance économique et sanitaire de la transmission de la cryptosporidiose chez les bovins. À ce jour, dix espèces de *Cryptosporidium* ont été détectées chez les bovins en Chine, avec un taux d’infection global de 11.9 %. Le taux d’infection le plus élevé (19.5 %) a été observé chez les veaux non sevrés, suivi par celui des juvéniles (10.69 %), des juvéniles sevrés (9.0 %) et des bovins adultes (4.94 %). Les espèces dominantes étaient *C. parvum* chez les veaux non sevrés et *C. andersoni* chez les bovins post-sevrés, juvéniles et adultes. Des espèces zoonotiques de *Cryptosporidium* (*C. parvum* et *C. hominis*) ont été trouvées chez les bovins, indiquant la possibilité de transmission entre l’homme et le bétail. Différentes races de bovins avaient des différences significatives dans le taux de prévalence et les espèces de *Cryptosporidium*. Cette synthèse a démontré que la distribution de *Cryptosporidium* est liée à l’âge, la race et la géographie, et fournit des références pour mieux comprendre les caractéristiques épidémiologiques et prévenir et contrôler la maladie.

## Introduction

Diarrhea is a common clinical symptom of various conditions and is harmful to animals. The causative agents include bacteria such as enterotoxigenic *Escherichia coli* (ETEC), viruses such as rotavirus, and parasites or other possible factors [10, 19]. *Cryptosporidium*, as an important protozoan parasite, can cause parasitic diarrhea in animals. This parasite has a broad distribution range in both developing and developed countries and can infect various hosts, including humans, domestic animals, and wildlife [26]. Infection with *Cryptosporidium* in cattle results in clinical symptoms such as diarrhea, abdominal pain, nausea, vomiting, and weight loss; however, such infections are generally not lethal [43]. Cattle, as a major domestic animal, can be infected by *Cryptosporidium*. Currently, *Cryptosporidium* infections in cattle are usually associated with four main species, i.e., *C. parvum*, *C. andersoni*, *C. ryanae*, and *C. bovis*. However, other species, including *C. suis*, *C. hominis*, *C. serpentis*, *C. xiaoi*, *C. ubiquitum*, *C. meleagridis*, *C. muris*, and *C. felis*, have also been identified in cattle [1, 3, 5, 6, 12, 13, 42, 48, 49].

The infection sites for different *Cryptosporidium* species vary and include the stomach, intestines, and respiratory tissues [36]. In cattle, *C. andersoni* mainly causes mucosal damage in the abomasums, whereas *C. parvum*, *C. ryanae*, and *C. bovis* usually result in villus atrophy, microvillus shortening, and destruction in the intestine [10, 15, 35]. *C. parvum* commonly infects humans as well as cattle, while *C. andersoni* and *C. bovis* have occasionally been reported in humans [40, 41]. Therefore, infected cattle are considered potentially important reservoirs of *Cryptosporidium* for human infections. A recent study demonstrated that zoonotic transmission may occur between cattle and farm workers due to close contact between cattle and humans [11, 33].

Although several studies have reported infections of cattle with *Cryptosporidium* species, there are no effective treatments and vaccines available for *Cryptosporidium* infection in China. Therefore, the purpose of this study was to determine the prevalence, genotypes, and subtypes of *Cryptosporidium* in China, evaluate age and breed-related differences in the incidence of this infection, and assess differences in the geographic distributions of *Cryptosporidium* species in China by reviewing a number of available published sources and data.

## Data sources and statistical analysis

We carried out a literature search without a language limitation in PubMed and the China National Knowledge Infrastructure (CNKI), covering all published papers until 2016, using a combination of the following keywords: *Cryptosporidium*, cattle, China. If an article in a language other than English was found, the abstract was screened, and the full text was reviewed to determine whether any additional information was included.

Chi-squared tests were used to compare *Cryptosporidium* infection rates, and differences with *p* values of less than 0.05 were considered significant.

## Results

### Prevalence of *Cryptosporidium* infection in cattle in different regions of China

In China, the first report of *Cryptosporidium* in cattle was published in 1986 in Lanzhou, which is located in Gansu Province [8]. According to the available published sources, *Cryptosporidium* species are distributed within 19 provinces in China, including northern China (Tianjin [31] and Inner Mongolia [52]), northeastern China (Heilongjiang [25, 55, 58]), eastern China (Shanghai [5, 59], Jiangsu [5], Anhui [5, 23, 51], Shandong [29], and Taiwan [46]), southern and central China (Henan [7, 16, 20, 24, 27, 29, 36, 44, 45], Hunan [29], Guangdong [47], and Guangxi [17, 50]), southwestern China (Sichuan [37] and Tibet [37]), and northwestern China (Gansu [37, 38, 56], Qinghai [2, 21, 28, 30, 32, 37, 54, 59], Ningxia [9, 18, 56], Xinjiang [14], and Shanxi [57]) (Tables 1 and 2). The overall infection rate was 11.9%, and infection rates varied significantly for different regions/provinces (*p* < 0.05). The regions with the highest infection rates were Taiwan, Inner Mongolia, Shandong, Hunan, and Qinghai. The regions with the lowest infection rates were Shanxi, Guangxi, Sichuan, Ningxia, and Gansu.

**Table 1.** Infection rates with *Cryptosporidium* in cattle in different regions of China.

Location	Sample no.	No. of positive samples	Infection rate (%)	Detection methods	Reference
Tianjin	136	11	8.09	a	[31]
Inner Mongolia	71	16	22.54	a	[52]
Heilongjiang	1483	257	17.33	a + b	[25, 55, 58]
Shanghai	497	67	13.48	a + b	[5, 59]
Jiangsu	1315	251	19.09	a + b	[5]
Anhui	1666	147	8.82	a, a + b	[5, 23, 51]
Shandong	148	36	24.3	b	[29]
Henan	4348	727	16.72	a, b, a + b	[7, 16, 20, 24, 25, 27, 34, 44, 45]
Hunan	181	43	23.8	b	[29]
Guangdong	1087	92	8.46	a	[47]
Guangxi	1438	36	2.50	a, a + b	[17, 50]
Sichuan	84	1	1.19	b	[37]
Tibet	44	4	9.09	b	[37]
Gansu	1450	69	4.76	b	[37, 38, 56]
Qinghai	2004	426	21.28	a, b, a + b, IFT + b	[2, 21, 28, 30, 32, 37, 53, 58]
Ningxia	3054	115	3.76	b, a + b	[18, 56]
Xinjiang	514	82	16.0	b	[14]
Shanxi	2071	70	3.4	b	[57]
Taiwan	460	173	37.6	a + b, IFT	[46]
Total	22051	2623	11.9		

*Notes.* a: by microscopy; b: by molecular methods; a + b: by microscopy and molecular methods; IFT: immunofluorescence test.

**Table 2.** Species and subtypes of *Cryptosporidium* in cattle in different regions of China.

Location	Identified species	Subtypes	Reference
Tianjin	*C. parvum*, *C. muris*		[31]*
Heilongjiang	*C. andersoni* (210), *C. bovis* (34), *C. parvum* (2), *C. ryanae* (6), *C. meleagridis* (5)	IIdA19G1 (1), IIIeA22G2R (3), A5A4A4A1 (5), A4A4A4A1 (33), A4A4A2A1 (2), A2A4A4A1 (2), A2A4A2A1 (1), A1A4A4A1 (2)	[25, 55, 58]
Anhui	*C. parvum*, *C. muris*		[5, 23, 51]*
Shandong	*C. andersoni* (11), *C. bovis* (13), *C. ryanae* (10)		[29]
Henan	*C. andersoni* (322), *C. bovis* (132), *C. parvum* (91), *C. ryanae* (30), *C. muris* (49), *C. ryanae + C. bovis* (11), *C. parvum + C. bovis* (6), *C. parvum + C. ryanae* (4), *C. parvum + C. andersoni* (3), *C. suis-like* (2)	IIdA19G1 (67)	[7, 18, 20, 24, 27, 29, 34, 44], [45]*
Hunan	*C. bovis* (7), *C. ryanae* (33)		[29]
Guangdong	*C. muris* (92)		[47]
Guangxi	*C. parvum*, *C. andersoni* (1)		*[17],[50]
Sichuan	*C. parvum* (1)		[37]
Tibet	*C. parvum* (4)	IIdA19G1 (1)	[37]
Gansu	*C. andersoni* (18), *C. bovis* (34), *C. parvum* (3), *C. ryanae* (13), *C. ubiquitum* (1)	IIdA15G1 (3), XIIa (1)	[37, 38, 56]
Qinghai	*C. andersoni* (3), *C. bovis* (91), *C. parvum* (21), *C. ryanae* (38), *C. ubiquitum* (1), *C. xiaoi* (1), *C. ryanae + C. bovis* (4), *C. parvum + C. bovis* (2), new *Cryptosporidium* genotype (4)	IIaA15G2R1 (8), IIaA16G2R1 (2), IIaA14G1R1 (1), IIaA14G2R1 (1), IIaA16G3R1 (1), IIdA15G1 (1)	[2, 21, 28, 30, 32, 37, 54, 59]
Ningxia	*C. andersoni* (23), *C. bovis* (45), *C. parvum* (34), *C. ryanae* (13)	IIdA15G1 (34)	[18, 56]
Xinjiang	*C. andersoni* (25), *C. bovis* (20), *C. parvum* (22), *C. ryanae* (9), *C. ryanae + C. bovis* (4), *C. parvum + C. bovis* (2)	IIdA14G1 (4), IIdA15G1 (11)	[14]
Shanxi	*C. andersoni* (70)	A4A4A4A1 (26), A1A4A4A1 (26), A2A4A4A1 (3), A4A4A2A1 (1)	[57]
Taiwan	*C. parvum* (173)		[46]
Total	*C. andersoni* (683), *C. bovis* (376), *C. parvum* (351) *C. ryanae* (152), *C. muris* (141), *C. ubiquitum* (2), *C. meleagridis* (5), *C. xiaoi* (1), *C. suis-lik*e (2), *C. ryanae + C. bovis* (19), *C. parvum + C. bovis* (10), *C. parvum + C. ryanae* (4), *C. parvum + C. andersoni* (3), New *Cryptosporidium* genotype (4)	IIaA14G1R1 (1), IIaA14G2R1 (1), IIaA15G2R1 (8), IIaA16G2R1 (2), IIaA16G3R1 (1), IIdA14G1 (4), IIdA15G1 (49), IIdA18G1 (1), IIdA19G1 (69), IIIeA22G2R1 (3), XIIa (1), A5A4A4A1 (5), A4A4A4A1 (59), A4A4A2A1 (3), A2A4A4A1 (5), A2A4A2A1 (2), A1A4A4A1 (28)	

* Study does not report the number of *Cryptosporidium* species separately.

More than 10 species of *Cryptosporidium*, including *C. andersoni*, *C. bovis*, *C. parvum*, *C. ryanae*, *C. muris*, *C. ubiquitum*, *C. meleagridis*, *C. xiaoi*, *C. suis-like*, mixed *Cryptosporidium* infection, and new *Cryptosporidium* genotypes, have been reported in cattle in China; the most common *Cryptosporidium* infections in cattle were caused by *C. bovis*, *C. parvum*, *C. ryanae*, and *C. muris*, whereas the other species were only found on occasion.

A variety of *Cryptosporidium* subtypes have been reported in China, including IIa subtypes (IIaA14G1R1, IIaA14G2R1, IIaA15G2R1, IIaA16G2R1, and IIaA16G3R1) and IId subtypes (IIdA14G1, IIdA15G1, IIdA18G1, and IIdA19G1) for *C. parvum*. Six *C. andersoni* subtypes were identified, including A5A4A4A1, A4A4A4A1, A4A4A2A1, A2A4A4A1, A2A4A2A1, and A1A4A4A1. The identified subtypes of *C. meleagridis* and *C. ubiquitum* were IIIeA22G2R1 and XIIa, respectively.

### Distributions of *Cryptosporidium* species/subtypes in cattle of different age groups in China

Cattle can be classified into four groups according to age: preweaned, postweaned, juvenile, and adult. The average infection rates in cattle differed according to age, ranging from 4.94% in adult cattle to 9.0%, 12.69%, and 19.5% in postweaned cattle, juvenile cattle, and preweaned cattle, respectively (*p* < 0.05; Table 3). Significant differences in average infection rates were noted among all age groups (*p* < 0.05). Previous studies in the USA have indicated that *C. parvum* is responsible for about 85–97% of *Cryptosporidium* infections in preweaned calves but only 1–4% of *Cryptosporidium* infections in postweaned calves and heifers [22]. The highest infection rates in each age group were 27.4% in adults, 28.8% in postweaned cattle, 31.7% in juvenile cattle, and 80% in preweaned cattle.

**Table 3.** Distribution of *Cryptosporidium* species/subtypes in cattle of different ages.

Age	No. specimens	No. positive	*Cryptosporidium* species (no.)	Subtype	Infection rate (%)	Reference
Preweaned	2734	533	*C. andersoni* (88), *C. bovis* (178), *C. parvum* (185), *C. ryanae* (50), *C. bovis* + *C. ryanae* (11), *C. parvum + C. bovis* (8), *C. parvum + C. ryanae* (4), *C. parvum + C. andersoni* (3), *C. meleagridis* (5)	IIdA14G1 (4); IIdA15G1 (92); IIdA19G1 (68); IIIeA22G2R1 (3); A4, A4, A4, A1 (3)	19.5	[1, 17, 23, 25, 30, 48, 52, 59]
Postweaned	3601	324	*C. andersoni* (231), *C. bovis* (59), *C. parvum* (15), *C. ryanae* (15), *C. bovis + C. ryanae* (3)	IIdA15G1 (2); A4, A4, A4, A1 (11); A2, A4, A4, A1 (1); A2, A4, A2, A1 (1); A1, A4, A4, A1 (10)	9.0	[1, 3, 17, 25, 29, 43, 52, 59]
Juveniles	2685	287	*C. andersoni* (214), *C. bovis* (45), *C. parvum* (3), *C. ryanae* (15), *C. xiaoi* (1), *C. suis-like* (2), *C. bovis + C. ryanae* (2)	A5A4, A4, A1 (5); A4, A4, A4, A1 (42); A4, A4, A2, A1 (3); A2, A4, A4, A1 (4); A2, A4, A2, A1 (1); A1, A4, A4, A1 (9)	10.69	[25, 28, 29, 32, 44, 56, 57, 58]
Adults	3196	158	*C. andersoni* (108), *C. bovis* (22), *C. ryanae* (18), *C. ubiquitum* (1), new *Cryptosporidium* genotype (2)	A4, A4, A4, A1 (3); A1, A4, A4, A1 (9)	4.94	[18, 25, 28, 29, 44, 56, 57]

The prevalence of specific *Cryptosporidium* species/subtypes was also varied among the different age groups of cattle. In preweaned cattle, *C. bovis* and *C. parvum* were the dominant *Cryptosporidium* species, and subtypes of IIdA14G1 [1], IIdA15G1 [22, 50, 57], IIdA19G1 [47], and IIIeA22G2R1 [47] were relatively common, with IIdA15G1 being the most prevalent. *C. andersoni* [1, 16, 24, 28, 47, 50], *C. ryanae* [1, 16, 24, 47, 50, 57], *C. meleagridis* [47], and mixed infection [1, 22, 24, 43] were also occasionally identified in preweaned cattle. In postweaned cattle, *C. andersoni* [1, 3, 16, 24, 28, 42, 50] was the most abundant species, and *C. bovis* [1, 16, 24, 28, 42, 50, 57], *C. parvum* [28, 50, 57], *C. ryanae* [1, 3, 24, 50], and mixed infection with *C. bovis* and *C. ryanae* [1] were rarely detected. Four subtypes of *C. andersoni* [16], characterized as A4A4A4A1, A1A4A4A1, A2A4A4A1, and A2A4A2A1, were also detected, whereas only one subtype (IIdA15G1) was identified for *C. parvum* [50]. The latter two subtypes for *C. andersoni* were considered the most prevalent. Juvenile cattle were found to be infected with *C. andersoni* [25, 28, 29, 44, 56–58], *C. bovis* [28, 29, 32], *C. parvum* [32], *C. ryanae* [28, 29, 32], *C. xiaoi* [28], *C. suis-like* [29], and mixed infection with *C. bovis* and *C. ryanae* [32]. The following *C. andersoni* [57, 58] subtypes were identified: A5A4A4A1, A4A4A4A1, A4A4A2A1, A2A4A4A1, A2A4A2A1, and A1A4A4A1. Adult cattle could be infected with *C. andersoni* [18, 25, 28, 29, 44, 56, 57], *C. bovis* [28, 29, 56], *C. ryanae* [28, 29], *C. ubiquitum* [28], and new genotypes [28]. No mixed *Cryptosporidium* infections were found in adult cattle. *C. andersoni* [57] formed two subtypes, i.e., A4A4A4A1 and A1A4A4A1.

In summary, *C. andersoni*, *C. bovis*, *C. ryanae*, and *C. parvum* were the most common *Cryptosporidium* species in cattle in China. *C. andersoni* was commonly found in postweaned, juvenile, and adult cattle, but had a relatively low prevalence in preweaned cattle. In contrast, *C. bovis* was mostly found in preweaned cattle. *C. ryanae* was more common in preweaned cattle than in cattle of other ages. *C. parvum* was mostly distributed in preweaned cattle.

### Distribution of *Cryptosporidium* species/subtypes in different cattle breeds in China

There are four main domesticated ungulate species in China, namely, dairy cattle, beef cattle, buffalo, and yaks. The prevalence of *Cryptosporidium* in different cattle breeds varied from 8.09% in beef cattle to 23.8% in buffalo (Table 4). The prevalence of *Cryptosporidium* in dairy cattle ranged from 1.68% to 47.68%, with an average infection rate of 10.44%. In yaks, the prevalence rate of *Cryptosporidium* infection ranged from 4% to 39.74%, with an average of 18.13%. In contrast, that in beef cattle ranged from 4.49% to 26.5%, with an average of 8.09%. The results of Chi-square tests showed that the prevalence differed significantly among the breed groups (*p* < 0.05). Moreover, the infection rates of dairy cattle were significantly different from those of beef cattle, buffalo, and yaks, with Chi-square values of 5.590, 33.347, and 108.509, respectively (*p* < 0.05). The differences between beef cattle and yaks, and between beef cattle and buffalo, were also statistically significant (*p* < 0.05). Several *Cryptosporidium* species, including *C. andersoni*, *C. bovis*, *C. parvum*, *C. ryanae*, *C. meleagridis*, *C. suis-like*, *C. parvum* (“mouse” genotype), *C. hominis*, *C. serpentis*, and mixed infection, have been reported in dairy cattle in China. *C. andersoni* was the dominant species in dairy cattle, and other species showed low infection rates. In dairy cattle, subtypes A4A4A4A1, A1A4A4A1, IIdA15G1, IIdA19G1, IIdA14G1, and IIIeA22G2R1 have been identified in China. Moreover, IIdA15G1 was the most common subtype of *C. parvum*, and A1A4A4A1 was the most common subtype of *C. andersoni*. In beef cattle, *C. andersoni*, *C. bovis*, *C. ryanae*, and mixed infection with *C. ryanae* and *C. bovis* were identified, with *C. andersoni* as the most prevalent species. In buffalo, *C. bovis* and *C. ryanae* infections have been reported. In yaks, *C. andersoni*, *C. bovis*, *C. parvum*, *C. ryanae*, *C. ubiquitum*, *C. xiaoi*, new *Cryptosporidium* genotypes, and mixed infection were found, with *C. bovis* having the highest prevalence, followed by *C. ryanae* and *C. parvum*. IIaA15G2R1 was the most endemic subtype, and IIaA14G1R1, IIaA14G2R1, IIaA16G2R1, IIaA16G3R1, IIdA15G1, IIdA18G1, IIdA19G1, and XIIa were also detected.

**Table 4.** Distribution of *Cryptosporidium* species/subtypes in dairy cattle, beef cattle, buffalo, and yaks.

Host	No. samples	No. positive	*Cryptosporidium* species (no.)	Subtype	Infection rate (%)	Reference
Dairy cattle	12743	1330	*C. andersoni* (475), *C. bovis* (321), *C. parvum* (165), *C. ryanae* (74), *C. meleagridis* (5), *C. suis-like* (2), *C. ryanae + C. bovis* (14), *C. parvum + C. bovis* (8), *C. parvum + C. ryana* (4), *C. parvum + C. andersoni* (3), *C. parvum ‘mouse’ genotype* (185), *C. hominis* (24), *C. serpentis* (4)	A4A4 A4A1 (1), A1A4 A4A1 (26), IIdA15G1 (97), IIdA19G1 (68), IIdA14G1 (4), IIIeA22G2R1 (3)	10.44	[5, 7, 9, 14, 18, 24, 25, 44, 45, 54–57]
Beef	1013	82	*C. andersoni* (53), *C. bovis* (16), *C. ryanae* (6), *C. ryanae + C. bovis* (1)	A4A4A4A1 (25), A2A4A4A1 (3), A2A4A2A1 (1), A4A4A2A1 (1)	8.09	[29, 57]
Buffalo	181	43	*C. bovis* (7), *C. ryanae* (33)		23.8	[29]
Yaks	2201	399	*C. andersoni* (3), *C. bovis* (96), *C. parvum* (28), *C. ryanae* (41), *C. ubiquitum* (2), *C. xiaoi* (1), new *Cryptosporidium* genotype (4), *C. bovis + C. ryanae* (4), *C. parvum + C. bovis* (2)	IIaA14G1R1 (1), IIaA14G2R1 (1), IIaA15G2R1 (8), IIaA16G2R1 (2), IIaA16G3R1 (1), IIdA15G1 (3), IIdA18G1 (1), IIdA19G1 (1), XIIa (1)	18.13	[2, 21, 29, 30, 32, 37, 38, 54, 59]

In summary, *C. andersoni* was the most common species of *Cryptosporidium* in beef cattle. *C. bovis* was identified as the predominant species responsible for yak infection, whereas *C. ryanae* was considered as the most prevalent in buffalo. *C. parvum* was more infectious to dairy cattle and yaks in China.

## Prevention and treatment

In developing countries, a major obstacle for disease control is the lack of effective methods to control *Cryptosporidium* infection and to decrease environmental contamination with oocysts [4]. In China, preventive hygiene measures and good management should be carried out to prevent the infection of cattle with *Cryptosporidium* spp. In calves, timely colostrum feeding is the simplest and most effective method to prevent diarrhea. For postweaned calves, the use of straw in pens and high-pressure cleaning has been shown to have preventive effects against contamination by *Cryptosporidium* oocysts [53].

The drugs used for the treatment of cryptosporidiosis include sulfadoxine-pyrimethamine, trimethoprim-sulfamethoxazole, quinacrine, pentamidine, bleomycin, elliptinium, alpha-difluoro-methylornithine, daunorubicin, and diclazuril. However, in an immunosuppressed rat model, none of these drugs were able to completely cure the disease [22]. Paromomycin and nitazoxanide are the only two drugs that have been analyzed in well-controlled clinical trials and have been shown to have some degree of efficacy [39]. More studies are needed to identify appropriate approaches to control *Cryptosporidium* infection and decrease contamination by oocysts in cattle farms.

## Conclusion

*Cryptosporidium* is widely distributed in cattle in China. Ten species have been identified and *C. andersoni*, *C. bovis*, *C. parvum*, and *C. ryanae* are the most common. Epidemiological analysis showed that there were significant differences in infection rates and species according to geography, age, and breed. In China, the highest infection rate was observed in preweaned cattle, the regions with high rates of infection were in eastern and northern China, while the most common *Cryptosporidium* species in cattle were *C. andersoni*, *C. bovis*, *C. ryanae*, and *C. parvum*. In addition, other factors, including examination methods and sample sizes (affecting the sensitivity and accuracy of the results), sanitation conditions (affecting the existence of *Cryptosporidium*), rearing conditions (influencing the health and immune status of cattle), and climate (influencing the survival of *Cryptosporidium* oocysts), may contribute to the occurrence of cryptosporidiosis. There are no effective treatments currently approved for this parasite, and preventive measures are difficult. For example, cattle owners should improve management, sanitation, and disinfection protocols and attempt to keep breeding houses clean and dry. Cattle should not be grazed in areas with a high occurrence of *Cryptosporidium*. Additionally, nutritional conditions should be optimized, and the government should aim to create awareness of the importance of hygiene promotion and reinforce support of *Cryptosporidium* research. The development of vaccines for this parasite may substantially improve outlooks.

Importantly, *C. parvum* and *C. hominis* in cattle may have zoonotic potential. People in affected areas should pay careful attention to hygiene. Additionally, more studies should be conducted to fully elucidate the pathogenesis and epidemiology of bovine Cryptosporidiosis. The findings of this study, which represent the first comprehensive analysis of *Cryptosporidium* prevalence in cattle in China, may contribute to a better understanding of the epidemiological features of *Cryptosporidium* in cattle.
